# T-Cell Traffic Jam in Hodgkin's Lymphoma: Pathogenetic and Therapeutic Implications

**DOI:** 10.1155/2011/501659

**Published:** 2010-10-12

**Authors:** Claudio Fozza, Maurizio Longinotti

**Affiliations:** Institute of Hematology, University of Sassari, Viale San Pietro 12, 07100 Sassari, Italy

## Abstract

In hematologic malignancies, the microenvironment is often characterized by nonneoplastic cells with peculiar phenotypic and functional features. This is particularly true in Hodgkin's lymphoma (HL), in which T lymphocytes surrounding Hodgkin's Reed-Sternberg cells are essentially polarized towards a memory T-helper type 2 phenotype. In this paper we will first evaluate the main processes modulating T-cell recruitment towards the lymph node microenvironment in HL, especially focusing on the role played by cytokines. We will then consider the most relevant mechanisms of immune escape exerted by neoplastic cells in order to evade antitumor immunity. The potential pathogenetic and prognostic impact of regulatory T cells in such a context will be also described. We will finally overview some of the strategies of cellular immunotherapy applied in patients with HL.

## 1. Infiltrating Lymphocytes in Hodgkin's Lymphoma

In hematologic malignancies, a specific pathogenetic role is often played by nonneoplastic cells within the microenvironment. This is particularly true in Hodgkin's lymphoma (HL), in which T lymphocytes surrounding Hodgkin's Reed-Sternberg cells (HRSCs) show peculiar phenotypic and functional features. A possible impairment of the T-cell compartment has been investigated in HL since the tuberculin purified protein derivative (PPD) skin test was found to be frequently negative in large series of patients, and therefore a dysregulation involving delayed cellular immune responses was hypothesized [1]. Although infiltrating T lymphocytes are essentially polarized towards a memory T-helper type 2 (Th2) phenotype [2], several studies have shown possible differences among the different subtypes of HL. Among the molecules potentially involved in modulating the pattern of T-cell infiltration, TARC (thymus and activation-regulated chemokine), a chemokine able to attract activated Th2 cells, was found on immunohistochemical staining to be highly expressed in most-classical HL, but not in lymphocyte-predominant HL and non-Hodgkin lymphomas (NHL). This different expression may at least partially justify the discrepancies observed among T-cell infiltrates in distinct HL subtypes [3]. Also, the T-cell transcription factor profile was shown to be consistent with the lymphoid compartment of origin of the different HL subtypes. In particular, in classical and nodular lymphocyte-predominant HL, the expression of GATA-3 and T-bet recalls the pattern typical of normal interfollicular T-cells and normal germinal centers, respectively [4]. Interestingly even the T-cell activation profile was compared in patients with lymphocyte-predominant HL with nodular or diffuse pattern. By evaluating CD134 and CD38 expression, it was shown that in the nodular variant, T cells express markers of transient/early T-cell activation, while the diffuse pattern is characterized by persistent cellular activation, resembling what was observed in T-cell-rich large B-cell NHL [5]. More recently, another report focused on lymphocyte-rich classical HL, showing that this subtype is characterized by a stronger expression of B-cell transcription factors in neoplastic cells and by a follicular T-cell background with features intermediate between the classical and the nodular lymphocyte-predominant variants [6]. 

The microarray technology was even able to highlight the prognostic significance of the composition of the T-cell infiltrate in a cohort of 267 patients. In fact, low numbers of infiltrating CD8+, CD56+, and CD57+ cells and high numbers of granzyme B+ and TIA-1+ cells were associated with a significantly worse outcome, as outlined by a higher frequency of leukocytosis, B symptoms, advanced clinical stage, and by lower response rates [7]. 

In order to shed some light on the mechanisms determining the composition of this microenvironment, the expression and function of T-cell homing molecules were specifically evaluated. Chemokine receptors: CXCR3, CXCR4, and CCR7 and adhesion molecules including CD62 ligand were found to be expressed on most T-cells within HL tissues, while the corresponding ligands were expressed on malignant cells and vascular endothelium. These features resemble the mechanisms of T cell recruitment observed in normal lymph nodes, thus further highlighting the cross talk among neoplastic and nonneoplastic cells within the HL microenvironment [8]. 

The issue of the clonality of infiltrating T lymphocytes was also assessed in two different studies. In the first one, CD8+ T cells were shown to be a polyclonal population with limited clonal expansion. In fact, although sequence analysis of the V regions revealed the presence of CD8+ expansions in all cases, most of these clonal expansions accounted for less than 10% of the whole CD8+ T cell population. Moreover, a comparison of the V region sequences did not provide evidence that CD8+ T-cells were driven towards a common antigen [9]. In the second study, the T cells adherent to HRSC were examined by single-cell analysis for the T cell receptor (TCR) gamma gene in 5 HL patients. While no evidence of a clonal expansion was obtained in four of five patients with classic HL, clonal TCR gamma sequences were detected in the only patient with lymphocyte-predominant HL, suggesting that even in HL patients, neoplastic cells may sometimes act as antigen-presenting cells [10]. 

A gene expression profile analysis was performed also on peripheral CD3+ T cells isolated from untreated patients with HL, showing that this cell subset seems to be involved in a Th1 immune response even in peripheral blood. However, this cell subpopulation, hypothetically supporting the anti-tumor immunity, appears to be functionally impaired, as shown by the balance in the expression of genes modulating either the cell cycle or the immune response [11]. 

## 2. Cytokine Production in the Hodgkin's Lymphoma Microenvironment

The release of cytokines within the lymph node microenvironment has been often advocated as possible mediator in the crosstalk among HL neoplastic cells and infiltrating T-cells. The overall pattern of cytokine gene expression has been shown to be similar in classic HL and reactive lymph nodes, as regards IL-2, IL-4, IL-5, and interferon (IFN)-gamma [12]. On the other side, it is known that HRSC can bind T lymphocytes in an antigen-independent way, based on LFA-3 (lymphocyte function-associated antigen 3) to CD2 and ICAM-1 (intercellular adhesion molecule 1) to LFA-1 interactions. Moreover, they show unusually strong expression of the costimulatory molecules CD80 and CD86, whose ligand CD28 is expressed on T-cells [13]. HRSC cells are also known to produce interleukin-(IL-) 1a, TNF-alpha, IL-6, IL-7, IL-9, IL-10, IL-13, and tumor growth factor-(TGF-) beta [14], the latter being characterized by a prominent role in suppressing T-cell responses [15]. 

More recently, the expression of potentially relevant chemokines and chemokine receptors was studied in lymph nodes from 24 patients with HL and in 5 control lymph nodes by combining in situ hybridization, immunohistochemistry, and flow cytometry. Among the different cytokines tested, RANTES was expressed almost exclusively by T cells. Among receptors, CCR3 and CCR5 were highly expressed in T cells—the former in both CD4+ and CD8+ cells, while the latter mainly in CD4+ cells. However, all these chemokines and receptors were not detected on neoplastic cells. This preferential distribution seems to suggest a potential but yet not completely explained involvement of these molecules in the leukocyte recruitment towards the HL lymph node [16]. The relationship between the expression on neoplastic cells of some specific CXC and CC chemokines such as MIG, IP10, and TARC and the infiltration of Th1 and Th2 lymphocytes was evaluated in another study, showing that all these chemokines may influence the Th2 polarization typical of HL [17].

Similarly, several interleukins with the relative receptors have been shown to play a role in the interaction among neoplastic and infiltrating cells. After the detection of the IL-3 receptor (IL-3R) on HRSC, it has been hypothesized that these neoplastic cells could be able to increase the production of IL-3 by preactivated T cells, thus suggesting an involvement of IL-3/IL-3R interactions in the neoplastic proliferation through a paracrine mechanism [18]. Moreover, both HL-derived cell lines and primary HRSC from lymph node tissues of HL patients express the IL-7 receptor. As these cell lines show the constitutive production of IL-7 and neutralizing anti-IL-7 antibodies induce a relevant inhibition of their basal proliferation, IL-7 seems to be also involved in autocrine circuitries modulating neoplastic cell growth. Moreover, the IL-7/IL-7R axis appears to act as a cofactor for the expansion of regulatory T-cells (Treg) and as a potential enhancer for the microenvironmental production of IL-6, a cytokine associated with the presence of “B” symptoms and poor outcome in HL [19]. Interestingly, in HL patients, also peripheral T lymphocytes have been shown to display an atypical profile of cytokine production, characterized by reduced intracellular IL-2, TNF-alpha, and IFN-gamma and increased cytoplasmic IL-4 production, being the CD3+CD8+ subpopulation specifically responsible for the increased levels of IL-4 [20, 21].

## 3. Mechanisms of Immune Escape by Neoplastic Cells

Considering that neoplastic cells are able to proliferate in HL although their microenvironment is characterized by an extensive inflammatory infiltrate, the mechanisms of immune escape have been deeply investigated in this disorder. For instance, one of the potentially involved molecules is the immunoregulatory glycan-binding protein galectin-1 (Gal1) which has been shown to be selectively overexpressed on HRSC. In fact, blockade of Gal1 was able to restore the Th1/Th2 balance which is usually inverted in HL, whereas treatment of activated T-cells with Gal1 favored the secretion of Th2 cytokines and the expansion of CD4+CD25 high forkhead box P3 (FOXP3)+ Treg. This finding therefore implies that Gal1 is involved in the development and maintenance of an immunosuppressive Th2/Treg-skewed microenvironment [22]. The role of this molecule was also examined in the context of Epstein-Barr virus-(EBV-) specific CD8+ T-cell responses in HL. Its expression was associated with a reduced CD8+ T-cell infiltration and more specifically with an impaired response towards latent membrane proteins (LMP) 1 and 2. Moreover, the in vitro exposure to recombinant Gal-1 inhibited proliferation and interferon-gamma expression by EBV-specific T cells [23]. 

Also the possible role played by the PD-1 (programmed death-1) protein was explored. In fact, this molecule and its ligand are thought to be involved in the functional impairment of T cells in chronic viral infections or tumor immune evasion. HL and T cell NHL, but not B-cell NHL, were shown to overexpress PD-1 ligand, while PD-1 was markedly elevated in tumor-infiltrating and peripheral T cells of HL patients. Moreover, blockade of the PD-1 system was able to restore the IFN-gamma production by HL-infiltrating T cells [24]. Using a genome-wide transcriptional approach, CD4+ T cells in HL but not in follicular NHL were demonstrated to be under the inhibitory influence of both TGF-beta and PD-1 *in vivo* [25]. An increase in the number of PD-1+ lymphocytes, measured within a tissue microarray platform, was also shown to be a stage-independent negative prognostic factor of overall survival as opposed to the number of FOXP3+ Treg [26]. All these findings seem to suggest that the impairment of the cellular immunity typical of HL can mirror a T cell exhaustion, which is at least partially mediated by the PD-1 signaling pathway. 

It is worth noting that very recently an increased number of tumor-associated macrophages was shown by gene-expression profiling to be significantly associated with primary treatment failure, shortened progression-free survival, increased likelihood of relapse after autologous hematopoietic stem cell transplantation, and shortened disease-specific survival in a large cohort of HL patients [27]. 

Several other molecules have been tested for their possible involvement in such a context. For instance, Prostaglandin E2 has been shown to impair CD4+ T-cell activation by interfering with the mechanisms of intracellular transduction downstream TCR and CD28 [28]. Another possible mechanism of T-cell activation impairment was identified in the reduced expression by HRSC of the B7 proteins [29]. Tissue inhibitor of metalloproteinases 1 (TIMP-1) is a protein with proteinase-inhibiting and cytokine properties which has been advocated not only as a survival factor for HRSC but, even more importantly, as potential immunosuppressive agent. In fact, *in situ* hybridization showed TIMP-1 RNA expression on HRSC, while TIMP-1 production by HRSC cells was demonstrated on immunohistochemical analysis. TIMP-1 was also shown to inhibit T cell cytotoxicity both against autologous cells presenting tumor-associated antigens and within allogeneic mixed lymphocyte cultures [30]. Also, the downregulatory molecule cytotoxic T lymphocyte-associated antigen 4 (CTLA-4) was shown to play a possible role, as the proportion of CTLA-4+/CD3+ cells negatively correlated with proliferative activity, IL-2 and IFN-gamma production by T lymphocytes in HL patients [31]. Even CD30, which is typically expressed on HRSC, was shown to inhibit T cell proliferation. More specifically, anti-CD3-stimulated T cells in the presence of CD30 failed to increase tritium uptake, to express CD25 and CD26 and to produce IL-2. This effect was however, reversed after addition of exogenous IL-2 or pretreatment of HRSC with anti-CD30 [32]. Interestingly, also the intracellular serpin proteinase inhibitor 9 (PI9) was found to be expressed in 10% of HL patients. This protein is the only known inhibitor of the proteolytic activity of granzyme B, the primary mediator of apoptosis induced by cytotoxic T lymphocytes and natural killer (NK) cells, thus suggesting another possible mechanism by which neoplastic cells could interfere with antitumor immunity in HL [33]. At least in other subtypes of lymphoma indoleamine 2,3-dioxygenase has been shown to be specifically, involved in the mechanisms of tumor resistance to chemoimmunotherapeutic treatments, likely due to an abnormally increased stimulation of tryptophan catabolism and to the consequent inhibition of antitumor immunity [34, 35].

## 4. Role of Regulatory T Cells

Treg represent a small fraction of peripheral CD4+ T cells which plays a crucial role in the maintenance of immune tolerance [36]. More specifically, they have been shown to influence the susceptibility to and the evolution of infective, neoplastic, and autoimmune diseases [37–39]. The possible involvement of T cells with immunomodulatory properties in HL pathogenesis was firstly hypothesized in 2004. Infiltrating T cells were shown to be anergic to different kinds of stimulation and to suppress peripheral mononuclear cells when cocultured. Furthermore, flow cytometry demonstrated a high frequency of both IL-10 and CD4+CD25+ Treg secretion in the lymph nodes of HL patients. Interestingly, their suppressive function was abrogated by IL-10 neutralization, prevention of cell-to-cell contactade and block of CTLA-4 [40]. One year later, some studies firstly suggested a possible positive prognostic impact for Treg in patients with HL. Expression of granzyme B, TIA-1, and FOXP3 was evaluated by immunohistochemistry in tissue microarrays of 257 patients, showing that low infiltration of Treg in association with high infiltration of cytotoxic T cells could predict an unfavorable outcome [41]. Similarly, a reduced FoxP3/granzyme B+ ratio was shown to predict poor failure free survival [42]. The absolute number of intratumoral FOXP3+ cells studied by immunohistochemistry on tissue microarrays was of independent prognostic significance for failure-free survival and of borderline significance for overall survival in classical HL [43]. All these findings, based on samples taken at diagnosis, were confirmed also in a cohort of patients with refractory or relapsed disease [44]. Only one study had results not in line with the above-mentioned data, showing that a high ratio of Treg over Th2 cells may result in a significantly shortened disease-free survival, therefore implying a possible inhibitory effect performed by Treg on antitumor immune responses [45]. 

Several mechanisms have been considered as possible mediators of Treg recruitment towards the HL microenvironment. For instance, the inhibition of CCR4 by using a chimeric anti-CCR4 monoclonal antibody was shown to deplete CCR4+ T cells and to inhibit the migration of CD4+CD25+ T cells *in vitro* [46]. Also, the aberrant expression of IL-21, apart from regulating STAT3 signaling and protecting HRSC from apoptosis, was demonstrated to attract Treg via regulation of macrophage-inflammatory protein-3alpha MIP-3alpha in HRSC [47]. The EBV infection was similarly considered as a potential mediator of Treg recruitment. In fact, the expression of the EBV nuclear antigen 1 in HRSC was shown to mediate the upregulation of the chemokine CCL20 and consequently the migration of Treg [48]. On the other side, some data seem to suggest that the expression of LAG-3 by tumor-infiltrating lymphocytes with regulatory properties could impair the anti-EBV CD8+ immune reaction in HL patients [49]. In another study, KM-H2, which was established as an HRSC line, was demonstrated to promote a bidirectional differentiation of CD4+ naive T cells toward Foxp3+ T-cells and CD4+ cytotoxic lymphocytes, suggesting that neoplastic cells may act within the HL microenvironment as antigen-presenting cells driving the differentiation of T helper cells [50].

The enrichment of Treg among infiltrating lymphocytes has been even suggested as a possible diagnostic marker, useful in distinguishing classical HL from other entities [51]. Increased frequencies of CD4+CD25+ Treg have also been demonstrated on peripheral blood of patients with HL [52]. More recently, apart from CD4+CD25+FoxP3+ Treg, other subfamilies of cells with immunomodulatory properties, such as CD4+CD26− T cells, have been suggested to potentially exert immunomodulatory functions within the lymph node microenvironment of patients with HL [53].

## 5. Strategies of Cellular Immunotherapy

Over the last few years, several studies have tried to establish if the biological impact that T lymphocytes have on HL pathogenesis could be translated into potential immunotherapeutic strategies. The demonstration, even in HL patients, of a graft-versus-lymphoma effect in the context of allogeneic hematopoietic stem cell transplantation and donor lymphocyte infusions [54], represents the ideal platform for such an approach. In order to overcome the apparent impairment of the antitumor immunity typical of HL, different mechanisms have been investigated. 

In particular, several laboratory studies have addressed the defective interaction among HRSC and T cells observed in course of HL. For instance, it is well known that HRSC preferentially attract Th2 T cells and Treg, therefore generating an immunosuppressed tumor environment. On the other side, CD8+ T cells lack CCR4 and are nonresponsive to chemokines usually expressed on HRSC. In this regard it was shown that a potential approach to make CD8+ T cells able to efficiently migrate towards HRSC was the induced expression of CCR4. Moreover, the concomitant expression of both CCR4 and of an anti-CD30 chimeric antigen receptor even allowed them to enhance tumor control when infused intravenously in mice engrafted with human HL [55]. Another potentially reversible feature of HRSC is the resistance to CD8+-mediated killing via granzyme B, at least partially due to defects in mitochondrial apoptotic pathways and elevated XIAP (X-linked inhibitor of apoptosis protein) expression. In fact, the expression of the proapoptotic factor Smac and the downregulation of XIAP by RNA interference were demonstrated to enhance the apoptotic response of HRSC as well as their susceptibility to CD8+ cytotoxicity [56]. Also, the stimulation of the zeta chain expression has been explored as potential immunotherapeutic approach in an in vitro study, as HL patients express on their peripheral T and NK cells reduced levels of this key molecule in TCR activation [57].

Another strategy designed to revert the functional anergy of T cells surrounding HRSC was based on a fusion protein represented by an anti-CD30 antibody targeting also IL-2, or IL-12. After binding to CD30+ HL cells, anti-CD30-IL-2 or anti-CD30-IL-12, antibody-cytokine fusion proteins were able to induce resting NK cells, but not T cells, to lyse the lymphoma cells with very high efficiency [58, 59]. Moreover, as TGF-beta secretion is able to inhibit tumor-specific cellular immunity, cytotoxic T lymphocytes have been engineered with a dominant negative TGF-beta receptor in order to resist the antiproliferative effects exerted by this molecule both *in vitro* and *in vivo* [60–62].

A very interesting point of view has been also offered by studies focusing on the role of the so-called side-population, constituted by progenitor cells within the neoplastic population with increased resistance to radiation and chemotherapy. It was shown that both HL cell lines and primary HL tumor samples contain a distinct side cell population, showing increased resistance to gemcitabine but also expressing higher levels of tumor-associated antigens, which could be potentially recognized and killed by specific cytotoxic T lymphocytes [63]. It is worth noting that even the pharmacologic effect of histone deacetylase inhibitors in HL may be associated with the induction of an antitumor activity and with a modulation of the cytokine and chemokine secretion within the lymph node microenvironment [64].

Lastly, cytokine-induced killer cells, that is, peripheral blood mononuclear cells expanded in the presence of IFN-gamma, IL-2, and a monoclonal antibody against CD3- have been infused in 7 patients with relapsed HL, showing some limited responses in few of them. Interestingly, these cells share functional and phenotypic properties with both T cells and NK cells and exhibit nonhuman leukocyte antigen (HLA-) restricted killing of tumor cell targets both *in vitro* and *in vivo* [65].

## 6. EBV Antigens as Potential Targets of Cytotoxic T Cells

Several studies have highlighted the pathogenetic role of the EBV infection in HL pathogenesis, thus implying that immunotherapeutic approaches could potentially rely on cytotoxic T-cells specifically directed towards EBV antigens. Interestingly, EBV expression seems to influence the composition of the infiltrating lymphocyte population, which on the other side may have an impact on clinical presentation and outcome [66]. 

As it is known that HLA class I molecules present viral peptides for recognition by CD8+ T cells and that this T cell subset plays a pivotal role in the control of EBV infection [67], several studies have focused on the possible impact on HL pathogenesis of the HLA-mediated EBV antigen presentation. Very interestingly, segments within the HLA class I locus have been demonstrated to be specifically associated with the susceptibility to EBV-positive HL, therefore suggesting that the antigenic presentation of EBV-derived peptides is somehow involved in the development of HL [68]. In another study by logistic regression, HLA-A*01 alleles were shown to be associated with increased and HLA-A*02 alleles with decreased risk of EBV-related HL. These allele-specific associations correspond to nearly 10-fold variation in the risk of HL between HLA-A*01 and HLA-A*02 homozygotes, pointing once again at the critical role of cytotoxic T-cell responses in EBV-related HL [69]. 

Interestingly, in the only case of spontaneous remission ever reported in HL, a specific impairment of the anti-EBV immunity was described at relapse [70]. The cytotoxic response to EBV latent antigens has been specifically characterized in both blood and tumor-infiltrating lymphocytes of HL patients. Actually EBV-specific CD8+ T cells were detected in blood and biopsy samples from both EBV-negative and EBV-positive patients. However, EBV-specific cytotoxic T-lymphocyte precursors were very rare in the blood of HL patients or even undetectable at the tumor site, further suggesting a possible impairment of the immune responses against EBV [71]. The existence of anti-EBV cytotoxic T cell responses in patients with EBV-negative HL was also confirmed in another study addressing the dynamic variance of this cell population during treatment [72].

The first relevant steps in the clinical setting were made in 2002 when EBV-specific cytotoxic T-lymphocytes were ex vivo generated in 13 patients with relapsed HL, by using autologous EBV-transformed B cells as stimulator cells. The infusion of these cytotoxic T-lymphocytes was followed by an increase in EBV-specific immunity, a decrease in virus load, homing and persistence of CD8+ T cells to sites of malignancy, resolution of constitutional symptoms, and some degree of tumor responses [73–75]. Even better results were obtained by exploiting genetically modified APCs in order to augment the expression and immunogenicity of LMP2 and, therefore, the frequency of LMP2-specific cytotoxic T lymphocytes [76]. Very interestingly, also allogeneic anti-EBV cytotoxic T cells have been applied in such a context, showing a good safety profile and once again some clinical responses [77]. Local delivery of IL-12 to tumor sites, by using EBV-specific T cells transduced with a retroviral vector expressing the p40 and p35 subunits of this cytokine, was explored as a possible strategy to overcome the inhibitory effects of the HL microenvironment on cytotoxic T lymphocytes [78].

As patients with active disease display a functional impairment of Ag-specific CD8+ T cells, some studies have focused on an improved targeting of these CD8+ T cells towards viral antigens expressed in HL. One of these strategies was based on the development of an adenoviral antigen presentation system able to reverse the functional T cell impairment and restore both IFN-gamma production and cytolytic function. With this approach, activated CD8+ T cells became able to respond to tumor cells and showed phenotypic features consistent with those of effector T cells [79]. One of the limits of immunotherapeutic approaches targeting EBV antigens is that a subset of malignant cells in the tumor may lack or lose the expression of these potential targets. EBV-specific cytotoxic T lymphocytes expressing a chimeric antigen receptor specific for CD30 have been designed in order to overcome this problem. These cells, generated from healthy donors and HL patients, were able to kill both EBV-positive and -negative cells and, in a xenograft model, produced antitumor effects against EBV−/CD30+ tumors [80].

## 7. Future Perspectives

Although HL is considered as one of the most curable hematologic malignancies, some relevant issues are yet to be resolved. Firstly, the prognosis of patients which poorly respond to or relapse after first-line treatments is still unsatisfactory. Moreover, identifying therapeutic approaches with minimal toxicity still looks essential in a disorder usually occurring in young adults. Studies further addressing the functional features of the T cell immune system in HL patients could not only offer a deeper understanding of the biology of this disease but, much more importantly, help to design new targeted immunotherapeutic strategies with increased specificity and therefore improved efficacy and toxicity profile.

## Abbreviations

CTLA-4: Cytotoxic T lymphocyte-associated antigen 4
EBV: Epstein-Barr virus
FOXP3: Forkhead box P3
Gal1: Galectin-1
HL: Hodgkin's lymphoma
HLA: Human leukocyte antigen
HRSC: Hodgkin's Reed-Sternberg cells
ICAM-1: Intercellular adhesion molecule 1
IFN: Interferon
IL: Interleukin
IL-3R: IL-3 receptor
LFA: Lymphocyte function-associated antigen
LMP: Latent membrane protein
NHL: Non-Hodgkin lymphomas
NK: Natural killer
PD-1: Programmed death-1
PPD: Purified protein derivative
TARC: Thymus and activation-regulated chemokine
TCR: T-cell receptor
TGF: Tumor growth factor
Th2: T-helper type 2
TIMP: Tissue inhibitor of metalloproteinases
Treg: Regulatory T-cells
XIAP: X-linked inhibitor of apoptosis protein.
